# Association of Sodium-Glucose Cotransporter–2 Inhibitors With Fracture Risk in Older Adults With Type 2 Diabetes

**DOI:** 10.1001/jamanetworkopen.2021.30762

**Published:** 2021-10-27

**Authors:** Min Zhuo, Chelsea E. Hawley, Julie M. Paik, Lily G. Bessette, Deborah J. Wexler, Dae H. Kim, Angela Y. Tong, Seoyoung C. Kim, Elisabetta Patorno

**Affiliations:** 1 Division of Pharmacoepidemiology and Pharmacoeconomics, Department of Medicine, Brigham and Women’s Hospital, Harvard Medical School, Boston, Massachusetts; 2 Division of Renal Medicine, Department of Medicine, Brigham and Women’s Hospital and Harvard Medical School, Boston, Massachusetts; 3 Division of Nephrology, Department of Medicine, Beth Israel Deaconess Medical Center, Harvard Medical School, Boston, Massachusetts; 4 New England Geriatric Research, Education and Clinical Center, VA Bedford Healthcare System, Bedford, Massachusetts; 5 New England Geriatric Research, Education and Clinical Center, VA Boston Healthcare System, Boston, Massachusetts; 6 Diabetes Center, Massachusetts General Hospital, Harvard Medical School, Boston; 7 Marcus Institute for Aging Research, Hebrew Senior Life, Harvard Medical School, Boston, Massachusetts; 8 Division of Gerontology, Department of Medicine, Beth Israel Deaconess Medical Center, Boston, Massachusetts; 9 Division of Rheumatology, Inflammation, and Immunity, Department of Medicine, Brigham and Women’s Hospital and Harvard Medical School, Boston, Massachusetts

## Abstract

**Importance:**

Whether sodium-glucose cotransporter–2 inhibitors (SGLT-2i) are associated with an increased risk of fractures in older adults with type 2 diabetes (T2D) outside of clinical trials remains unknown.

**Objective:**

To examine the association of incident fracture among older adults with T2D with initiating an SGLT-2i compared with initiating a dipeptidyl peptidase 4 inhibitor (DPP-4i) or a glucagon-like peptide 1 receptor agonist (GLP-1RA).

**Design, Setting, and Participants:**

This is a population-based, new-user cohort study including older adults (aged ≥65 years) with T2D enrolled in Medicare fee-for-service from April 2013 to December 2017. Data analysis was performed from October 2020 to April 2021.

**Exposures:**

New users of an SGLT-2i, DPP-4i, or GLP-1RA without a previous fracture were matched in a 1:1:1 ratio using 3-way propensity score matching.

**Main Outcomes and Measures:**

The primary outcome was a composite end point of nontraumatic pelvic fracture, hip fracture requiring surgery, or humerus, radius, or ulna fracture requiring intervention within 30 days. After 3-way 1:1:1 propensity score matching, multivariable Cox proportional hazards regression models were used to generate hazard ratios (HRs) for SGLT-2i compared with DPP-4i and GLP-1RA and Kaplan-Meier curves to visualize fracture risk over time across groups.

**Results:**

Of 466 933 new initiators of study drugs, 62 454 patients were new SGLT-2i users. After 3-way matching, 45 889 (73%) new SGLT-2i users were matched to new users of DPP-4i and GLP-1RA, yielding a cohort of 137 667 patients (mean [SD] age, 72 [5] years; 64 126 men [47%]) matched 1:1:1 for analyses. There was no difference in the risk of fracture in SGLT-2i users compared with DPP-4i users (HR, 0.90; 95% CI, 0.73-1.11) or GLP-1RA users (HR, 1.00; 95% CI, 0.80-1.25). Results were consistent across categories of sex, frailty (nonfrail, prefrail, and frail), age (<75 and ≥75 years), and insulin use (baseline users and nonusers).

**Conclusions and Relevance:**

In this nationwide Medicare cohort, initiating an SGLT-2i was not associated with an increased risk of fracture in older adults with T2D compared with initiating a DPP-4i or GLP-1RA, with consistent results across categories of frailty, age, and insulin use. These findings add to the evidence base evaluating the potential risks associated with SGLT-2i use for older adults outside of randomized clinical trials.

## Key Points

**Question:**

Are sodium-glucose cotransporter–2 inhibitors (SGLT-2i) associated with increased risk of fracture in older adults with type 2 diabetes?

**Findings:**

In this nationwide cohort study of 137 667 Medicare beneficiaries aged 65 years or older with type 2 diabetes without a previous fracture, after 1:1:1 propensity score matching, there was no difference in fracture risk among new users of SGLT-2i compared with users of dipeptidyl peptidase 4 inhibitors or glucagon-like peptide 1 receptor agonists. Results were consistent across categories of sex, frailty, age, and insulin use.

**Meaning:**

The initiation of SGLT-2i was not associated with an increased risk of fracture in older adults with type 2 diabetes compared with other diabetes agents, and these findings add to the evidence base evaluating the safety profile of SGLT-2i in older adults.

## Teaser

This cohort study of older adults with type 2 diabetes examines the association of incident fracture with initiating a sodium-glucose cotransporter–2 inhibitor compared with initiating a dipeptidyl peptidase 4 inhibitor or a glucagon-like peptide 1 receptor agonist.

## Introduction

Older adults with type 2 diabetes (T2D) are at an increased risk of death from cardiovascular disease compared with older adults without T2D.^1^ Sodium-glucose cotransporter–2 inhibitors (SGLT-2i) are oral diabetes medications that reduce the risk of atherosclerotic cardiovascular events, hospitalization for heart failure, end-stage kidney disease, and death among adults with T2D.^2,3,4,5,6^ There is, however, a concern that SGLT-2i may be associated with an increased risk of fracture on the basis of findings in 1 randomized clinical trial (RCT).^7^ Together, T2D and aging may have negative effects on bone metabolism.^8,9,10^ In addition, other comorbidities, such as osteoporosis and chronic kidney disease, also increase the risk of fracture in older adults.^11,12,13^ Thus, understanding the fracture risk associated with SGLT-2i in older adults with T2D is critical.

SGLT-2i lower blood glucose levels by promoting urinary glucose excretion.^14^ SGLT-2i also augment urinary phosphate reabsorption, triggering the parathyroid hormone and fibroblast growth factor 23; this action has the potential to harm bone health.^15,16^ In the Canagliflozin Cardiovascular Assessment Study (CANVAS), the incidence rate (IR) of bone fractures among those taking canagliflozin, an SGLT-2i, was significantly higher than that among those taking placebo (15.4 vs 11.9 fractures per 1000 person-years; hazard ratio [HR], 1.26; 95% CI, 1.04-1.52).^3^ This increased risk of fracture, however, was not observed in other large RCTs of canagliflozin or other SGLT-2i.^2,4,5,6,17^ Fewer than one-half of participants in these RCTs were adults aged 65 years and older, leading to a lack of data on fracture incidence in older adults taking any SGLT-2i.^2,4,5,6,17^ The present study sought to determine whether taking any SGLT-2i vs other diabetes agents is associated with an increased risk of fracture for older adults.

## Methods

### Study Design and Data Sources

We performed a population-based, new-user cohort study using Medicare fee-for-service data. Medicare is a nationwide US federal health insurer for eligible individuals primarily aged 65 years and older that provides coverage for inpatient and outpatient services and prescription medications. We leveraged Medicare claims data from Parts A, B, and D, including dates and place of service, *International Classification of Diseases, Ninth Revision, Clinical Modification* (*ICD-9-CM*) and *International Statistical Classification of Diseases, Tenth Revision, Clinical Modification* (*ICD-10-CM*) codes, *Current Procedural Terminology, Fourth Edition* codes, type of clinician, National Drug Codes, and prescription drug days supplied. This study was approved by the Brigham and Women’s institutional review board, and an appropriate data use agreement was in place. Informed consent was not obtained because the study used a Medicare administrative dataset of claims data with anonymous identifiers, in accordance with 45 CFR §46. This report followed the Strengthening the Reporting of Observational Studies in Epidemiology (STROBE) reporting guideline for observational studies.

### Study Population

We included patients aged 66 years and older with T2D who were newly prescribed an SGLT-2i, dipeptidyl peptidase 4 inhibitor (DPP-4i), or glucagon-like peptide 1 receptor agonist (GLP-1RA) between April 1, 2013 (after the first SGLT-2i was approved in the US), and December 31, 2017. We set the age threshold to 66 years at cohort entry so that patients would have at least 1 year of Medicare eligibility before cohort entry. The cohort entry date was the day of the first prescription claim date during our study period. Eligible patients must have had at least 365 days of Medicare Parts A, B, and D enrollment before cohort entry. We excluded patients with prior use of any of the 3 medication of interest in the 365-day covariate assessment period, as well as those who received more than 1 medication of interest on the cohort entry date. We excluded patients admitted to a nursing home 365 days before the index date because we may not have been able to determine whether they received a study medication of interest during the admission. Because we were interested in studying incident fracture events, patients were excluded if they had an inpatient or outpatient *ICD-9-CM* or *ICD-10-CM *diagnostic code for a previous fracture (ie, pelvis, hip, humerus, radius, or ulna) during the covariate assessment period. We also excluded those who had an inpatient or outpatient *ICD-9-CM* or *ICD-10-CM* or *Current Procedural Terminology, Fourth Edition* code for any of the following during the covariate assessment period: type 1 diabetes, non–skin cancer, human immunodeficiency virus, or end-stage kidney disease (dialysis or prior renal transplant) (eTable 1 in the Supplement). Individuals meeting the inclusion criteria could contribute to each cohort only once.

### Exposures

The primary exposure of interest was new use of any SGLT-2i (canagliflozin, dapagliflozin, or empagliflozin), DPP-4i (alogliptin, linagliptin, saxagliptin, or sitagliptin), or GLP-1RA (albiglutide, dulaglutide, exenatide, liraglutide, lixisenatide, or semaglutide). We identified new users of medications of interest through claims for filled prescriptions. We chose these 2 active comparator diabetes agents, DPP-4i and GLP-1RA, which could be chosen as second-line therapies for T2D, as similarly positioned in the treatment algorithm of patients with T2D.^18^

### Outcomes and Follow-up

The primary outcome was a composite of nontraumatic pelvic fracture, hip fracture requiring surgery, or humerus, radius, or ulna fracture requiring intervention within 30 days (eTable 2 in the Supplement). Algorithms based on claims data to identify these fractures have been previously validated with a positive predictive value greater than 92%.^19,20,21^ Secondary outcomes included incidence of falls, hypoglycemia,^22^ and syncope. We also validated our findings against 2 positive control outcomes: diabetic ketoacidosis and heart failure hospitalization rates.^23^ Prior studies have shown that SGLT-2i are associated with a significantly higher risk of diabetic ketoacidosis^24,25^ and significantly lower risk of heart failure hospitalization compared with DPP-4i and GLP-1RA.^26,27,28^

Patients contributed person-time from the day after cohort entry until the occurrence of any of the following: death; end of health care or pharmacy enrollment; starting or stopping an SGLT-2i, DPP-4i, or GLP-1RA during follow up; end of study data; or occurrence of a study outcome. We considered medications as discontinued if there was more than a 60-day period between prescription claims for the medication of interest. We considered patients at risk for an event for 60 days after their last prescription of the medication of interest should have run out.^22^

### Covariates

We assessed patient demographic characteristics in the 365-day period before cohort entry through the index date. Baseline covariates were selected on the basis of previous studies and clinical expertise.^22,29^ Data included codes for diabetes-related comorbidities, comorbid conditions, fall- or fracture-related conditions and medications, other medications, and health care utilization. We used the Claims-based Frailty Index^30,31^ to estimate frailty. The index is a continuous scale from 0 to 1, with higher numerical values indicating more frailty. We categorized frailty into 3 groups using the cutpoints of less than 0.15 (nonfrail), 0.15 to 0.24 (prefrail), and greater than or equal to 0.25 (frail).^30,31,32,33^ We also included claims for laboratory monitoring and screenings, such as hemoglobin A_1c_ and bone mineral density screening (see eTable 3 in the Supplement for the full list). Stratification variables for secondary analyses included sex, frailty status, age, and insulin use vs nonuse.

### Statistical Analysis

To mitigate the risk of confounding by indication, we used 3-way propensity matching at a ratio of 1:1:1 to create 3 groups of patients initiating SGLT-2i, DPP-4i, or GLP-1RA with balanced covariates.^34^ The 1:1:1 propensity score–matched cohort was created using nearest-neighbor matching within a maximum caliper width of 0.05. The final 3-way matched cohort is expected to include individuals with similar observed characteristics overall and a roughly equal likelihood of receiving each of the 3 drugs of interest. We assessed covariate balance among the matched cohorts by using standardized differences: standardized differences less than 0.1 suggest negligible differences between matched groups.^35,36^ We matched on 58 covariates (eTable 3 in the Supplement).

After 3-way matching, we generated multivariable Cox proportional hazards regression models and evaluated the IR of fracture per 1000 person-years. All models were conducted in propensity score-matched groups and did not include any terms other than exposure group. Effect estimates were HRs with 95% CIs. We generated Kaplan-Meier curves to visualize the cumulative incidence of fracture events over time. We compared fracture incidence in each group using the log-rank test with a 2-sided significance threshold of *P* < .05. Analyses were conducted using the Aetion Evidence Platform software for real-world data analysis version 2020 (Aetion, Inc)^37^ and SAS statistical software version 9.4 (SAS Institute, Inc). Data analysis was performed from October 2020 to April 2021.

We performed several sensitivity analyses on our primary outcome to assess the robustness of the study findings. We considered death as a competing risk for fracture using the Fine and Gray method.^38,39^ We also changed the grace period from 60 to 30 and 90 days. To address potential informative censoring, we carried forward the exposure to the index medication for 365 days without considering drug discontinuation or switching to mimic an intention-to-treat approach.^22^ We did not carry the exposure beyond 365 days to minimize misclassification of the exposure. Finally, because canagliflozin was the SGLT-2i previously associated with increased risk of fracture,^3^ we reevaluated the risk of fracture specifically among patients initiating canagliflozin (64% of SGLT-2i group) compared with patients initiating a DPP-4i or GLP-1RA.

We tested for the presence of effect modification in 4 relevant subgroups: (1) female and male sex; (2) nonfrail, prefrail, and frail patients^30,31,32^; (3) age less than 75 years vs 75 years or older, and (4) baseline insulin users vs nonusers. For each analysis, we ran a new propensity score match within each subgroup and then ran a Cox proportional hazard model within each matched subgroup. Outcome models did not include any terms other than exposure group.

## Results

### Study Population

**Figure 1.**
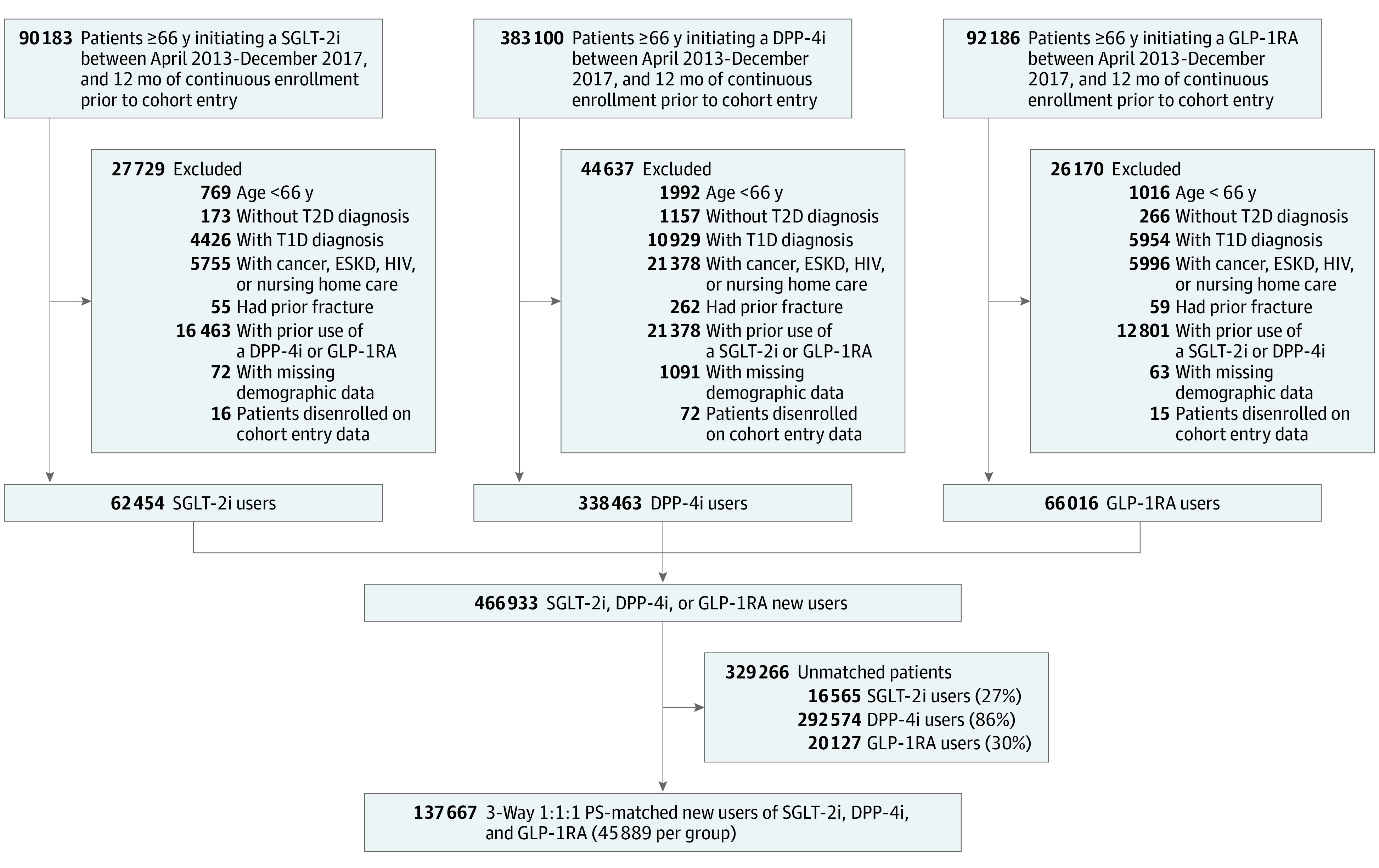
Flow Diagram of the Study Population We included patients aged 66 years and older with type 2 diabetes (T2D) who were newly prescribed a sodium-glucose cotransporter–2 inhibitor (SGLT-2i), dipeptidyl peptidase 4 inhibitor (DPP-4i), or glucagon-like peptide 1 receptor agonist (GLP-1RA) between April 1, 2013 (after the first SGLT-2i was approved in the US), and December 31, 2017. We set the age threshold to 66 years at cohort entry so that patients would have at least 1 year of Medicare eligibility before cohort entry. A total of 466 933 patients met the study inclusion and exclusion criteria: 62 454 (13%) SGLT-2i new users, 338 463 (73%) DPP-4i new users, and 66 016 (14%) GLP-1RA new users. After 1:1:1 3-way propensity score matching, we identified 45 889 matched sets of patients initiating SGLT-2i, DPP-4i, or GLP-1RA, for a total of 137 667 patients: 73% of SGLT-2i users were matched. Demographic information includes age, sex, race, and region. ESKD indicates end-stage kidney disease; PS, propensity score; T1D, type 1 diabetes.

A total of 466 933 patients met the study inclusion and exclusion criteria: 62 454 (13%) SGLT-2i new users, 338 463 (73%) DPP-4i new users, and 66 016 (14%) GLP-1RA new users (Figure 1). SGLT-2i initiators had lower prevalence of comorbid conditions and were less likely to be frail (6651 participants [10.65%]) compared with DPP-4i initiators (60 005 participants [17.73%]) and GLP-1RA initiators (10 768 participants [16.31%]); DPP-4i initiators were older (mean [SD] age, 74.69 [6.71] years) than SGLT-2i initiators (mean [SD] age, 71.94 [5.17] years) and GLP-1RA users (mean [SD] age, 71.46 [4.84] years); and GLP-1RA users had more frequent insulin use (29 693 participants [44.98]) than SGLT-2i initiators (17 492 participants [28.01]) (Table 1 and eTable 3 in the Supplement).

**Table 1.** Selected Baseline Characteristics in the SGLT-2i, DPP-4i, and GLP-1RA Groups After Propensity Score Matching

Baseline characteristics	Patients, No. (%)					
	Unmatched			3-Way propensity score–matched^a^		
	SGLT-2i (n = 62 454)	DPP-4i (n = 338 463)	GLP-1RA (n = 66 016)	SGLT-2i (n = 45 889)^b^	DPP-4i (n = 45 889)	GLP-1RA (n = 45 889)
Demographic						
Age, mean (SD), y	71.94 (5.17)	74.69 (6.71)	71.46 (4.84)	71.60 (4.96)	71.64 (5.13)	71.67 (4.97)
Sex						
Female	30 658 (49.09)	190 686 (56.34)	36 952 (55.97)	24 341 (53.04)	24 836 (54.12)	24 364 (53.1)
Male	31 796 (50.91)	147 777 (43.66)	29 064 (44.03)	21 548 (46.96)	21 053 (45.88)	21 525 (46.9)
Race and ethnicity						
Black	4577 (7.33)	35 969 (10.63)	5515 (8.35)	3534 (7.70)	3484 (7.59)	3586 (7.81)
Other^c^	6457 (10.34)	48 256 (14.26)	4871 (7.38)	3729 (8.13)	3701 (8.07)	3848 (8.39)
White	51 420 (82.33)	254 238 (75.12)	55 630 (84.27)	38 626 (84.17)	38 704 (84.34)	38455 (83.80)
Region						
Midwest	12 732 (20.39)	70 357 (20.79)	15 625 (23.67)	10 197 (22.22)	10 215 (22.26)	10 109 (22.0)
Northeast	10 715 (17.16)	63 919 (18.89)	10 113 (15.32)	7330 (15.97)	7158 (15.60)	7349 (16.01)
South	28 117 (45.02)	143 629 (42.44)	28 648 (43.40)	20 391 (44.44)	20 541 (44.76)	20 416 (44.4)
West	10 890 (17.44)	60 558 (17.89)	11 630 (17.62)	7971 (17.37)	7975 (17.38)	8015 (17.47)
Diabetes-related conditions						
Nephropathy	6488 (10.39)	48 370 (14.29)	12 119 (18.36)	5944 (12.95)	6038 (13.16)	6105 (13.30)
Neuropathy	15 808 (25.31)	79 491 (23.49)	21 058 (31.90)	12 859 (28.02)	13 111 (28.57)	12 966 (28.2)
Retinopathy	6513 (10.43)	32 838 (9.70)	8972 (13.59)	5294 (11.54)	5306 (11.56)	5323 (11.60)
Endocrinologist visit during prior 365 d	10 894 (17.44)	44 589 (13.17)	18 034 (27.32)	9732 (21.21)	9541 (20.79)	9960 (21.70)
Hemoglobin A_1c_ tests ordered during prior 365 d, mean (SD), No.	2.75 (1.33)	2.60 (1.40)	2.84 (1.41)	2.79 (1.35)	2.78 (1.39)	2.77 (1.36)
Hypoglycemia	4871 (7.80)	26 451 (7.82)	6342 (9.61)	3845 (8.38)	3921 (8.54)	3974 (8.66)
Comorbid conditions						
Heart failure	7037 (11.27)	55 737 (16.47)	10 235 (15.50)	5825 (12.69)	5965 (13.00)	5979 (13.03)
Hypertension	57 598 (92.22)	315 528 (93.22)	61 841 (93.68)	42 626 (92.89)	42 657 (92.96)	42 650 (92.9)
Ischemic heart disease	21 178 (33.91)	120 679 (35.66)	23 866 (36.15)	15 834 (34.51)	15 788 (34.40)	15 914 (34.6)
Ischemic or hemorrhagic stroke	7231 (11.58)	47 627 (14.07)	8116 (12.29)	5418 (11.81)	5504 (11.99)	5429 (11.83)
Renal disease (nondiabetic)	11 987 (19.19)	107 932 (31.89)	20 177 (30.56)	10 848 (23.64)	10 955 (23.87)	10920 (23.80)
Falls or fracture-related conditions						
Bone mineral density screening	4864 (7.79)	27 645 (8.17)	5941 (9.00)	3916 (8.53)	3939 (8.58)	3911 (8.52)
Dementia	3573 (5.72)	35 947 (10.62)	4023 (6.09)	2664 (5.81)	2856 (6.22)	2735 (5.96)
Falls or syncope	3373 (5.40)	26 349 (7.78)	4489 (6.80)	2777 (6.05)	2837 (6.18)	2777 (6.05)
Frailty category^d^						
Nonfrail	19 689 (31.53)	82 847 (24.48)	14 905 (22.58)	12 319 (26.85)	11 976 (26.10)	12 183 (26.5)
Prefrail	36 114 (57.82)	19 5611 (57.79)	40 343 (61.11)	27 818 (60.62)	27 940 (60.89)	27 779 (60.5)
Frail	6651 (10.65)	60 005 (17.73)	10 768 (16.31)	5752 (12.53)	5973 (13.02)	5927 (12.92)
Glaucoma or cataracts	27 040 (43.30)	140 445 (41.49)	28 931 (43.82)	19 989 (43.56)	19 997 (43.58)	19965 (43.51)
Mobility limitations	1726 (2.76)	15 564 (4.60)	2461 (3.73)	1379 (3.01)	1433 (3.12)	1433 (3.12)
Osteoporosis	4458 (7.14)	33 373 (9.86)	4932 (7.47)	3305 (7.20)	3375 (7.35)	3402 (7.41)
Falls or fracture-related medications						
Angiotensin converting enzyme inhibitors or angiotensin-receptor blockers	48 725 (78.02)	261 515 (77.27)	52 582 (79.65)	36 259 (79.01)	36 190 (78.86)	36234 (78.96)
Anticholinergics	12 271 (19.65)	73 955 (21.85)	15 032 (22.77)	9687 (21.11)	9901 (21.58)	9679 (21.09)
Anticonvulsants	12 586 (20.15)	67 714 (20.01)	16 649 (25.22)	10 331 (22.51)	10 646 (23.20)	10 362 (22.5)
Antidepressants	17 786 (28.48)	93 185 (27.53)	23 568 (35.70)	14 854 (32.37)	15178 (33.08)	15 055 (32.8)
Benzodiazepines	5205 (8.33)	30 516 (9.02)	6051 (9.17)	4087 (8.91)	4060 (8.85)	22 863 (49.8)
β-blockers	30 249 (48.43)	175 935 (51.98)	34 252 (51.88)	22 732 (49.54)	22 791 (49.67)	15 589 (33.9)
Calcium channel blockers	20 700 (33.14)	128 587 (37.99)	23 149 (35.07)	15 595 (33.98)	15 450 (33.67)	10029 (21.85)
Diuretics						
Loop	11 326 (18.13)	77 999 (23.05)	17 758 (26.90)	9789 (21.33)	10 081 (21.97)	9997 (21.77)
Thiazide	9860 (15.79)	56 419 (16.67)	12 036 (18.23)	7751 (16.89)	7920 (17.26)	7833 (17.07)
Other	2614 (4.19)	16 240 (4.80)	3997 (6.05)	2262 (4.93)	2284 (4.98)	2297 (5.01)
Nitrates	5859 (9.38)	36 423 (10.76)	7089 (10.74)	4470 (9.74)	4425 (9.64)	4500 (9.81)
Opioids	9217 (14.76)	55 285 (16.33)	12 474 (18.90)	7608 (16.58)	7830 (17.06)	7730 (16.84)
Osteoporosis medications^e^	2445 (3.91)	20 643 (6.10)	2483 (3.76)	1718 (3.74)	1761 (3.84)	1735 (3.78)
Sedative hypnotics^f^	2452 (3.93)	14 485 (4.28)	3100 (4.70)	1970 (4.29)	1909 (4.16)	1965 (4.28)
Oral steroids	11 763 (18.83)	65 136 (19.24)	13 447 (20.37)	8966 (19.54)	8943 (19.49)	9081 (19.79)
Total medications, mean (SD), No.	12.92 (5.82)	13.13 (6.10)	14.54 (6.19)	13.60 (5.99)	13.71 (6.14)	13.72 (5.82)
Diabetes medications						
Diabetes drugs, mean (SD), No.	2.34 (0.80)	2.18 (0.77)	2.32 (0.82)	2.32 (0.81)	2.33 (0.81)	2.33 (0.82)
Insulin	17 492 (28.01)	52 226 (15.43)	29 693 (44.98)	16 312 (35.55)	16 234 (35.38)	16403 (35.74)
Metformin	48 832 (78.19)	247 812 (73.22)	44 883 (67.99)	33 971 (74.03)	34 081 (74.27)	33932 (73.94)
Sulfonylureas	29 342 (46.98)	164 362 (48.56)	27 694 (41.95)	20 083 (43.76)	20 746 (45.21)	20614 (44.92)
Thiazolidinediones	6766 (10.83)	29 792 (8.80)	6547 (9.92)	4682 (10.20)	4755 (10.36)	4868 (10.61)
Healthcare utilization						
Emergency department visits during prior 365 d	16 498 (26.42)	11 4540 (33.84)	20 435 (30.95)	12 996 (28.32)	13 263 (28.90)	13 152 (28.64)
Hospitalization during prior 365 d	7401 (11.85)	62839 (18.57)	9829 (14.89)	5930 (12.92)	6165 (13.43)	6072 (13.22)
Office visits during prior 365 d, mean (SD), No.	10.88 (7.55)	11.18 (8.20)	12.51 (8.56)	11.53 (7.85)	11.58 (8.27)	4028 (8.78)

Abbreviations: DPP-4i, dipeptidyl peptidase inhibitors; GLP-1RA, glucagon-like peptide-1 receptor agonists; SGLT-2i, sodium-glucose cotransporter–2 inhibitors.

^a^ All standardized differences between the 3 drugs in each polypharmacy group were less than 0.10, indicating well-balanced groups after propensity score matching.

^b^ A total of 29 396 patients (64%) in the matched SGLT-2i group were new canagliflozin users.

^c^ Race and ethnicity information were taken directly from Medicare data input. Other race and ethnicity includes race and ethnicity indicated specifically as Asian, Hispanic, North American Native, other, or unknown.

^d^ The Claims-based Frailty Index^30,31,32,33^ was used to estimate frailty. The index is a continuous scale from 0 to 1, with higher numerical values indicating more frailty. Nonfrail is defined as a score less than 0.15, prefrail is a score of 0.15 to 0.24, and frail is a score of 0.25 or higher.

^e^ Osteoporosis medications included bisphosphonates, calcitonin, denosumab, raloxifene, romosozumab, tamoxifen, and teriparatide.

^f^ Sedative hypnotics included buspirone, chloral hydrate, diphenhydramine, doxylamine, eszopiclone, hydroxyzine, mepbromate, zaleplon, and zolpidem.

After 1:1:1 3-way propensity score matching, we identified 45 889 matched sets of patients initiating SGLT-2i (73% of SGLT-2i users), DPP-4i, or GLP-1RA, for a total of 137 667 patients (mean [SD] age, 72 [5] years; 64 126 men [47%]). Of these, 29 396 participants (64%) were taking canagliflozin. After matching, all covariates were balanced, with standardized differences less than 0.1 (Table 1). The median duration of follow-up for the fracture outcome was 262 days in the SGLT-2i group, 278 days in the DPP-4i group, and 249 days in the GLP-1RA group (eTable 4 in the Supplement).

### Primary Fracture Outcome

**Figure 2.**
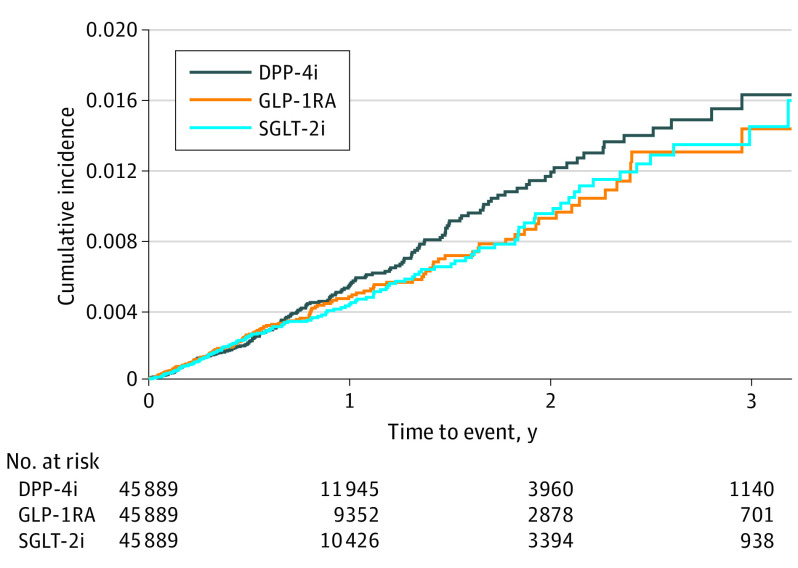
Kaplan-Meier Curves for Incidence of Fractures Within Matched Groups The cumulative incidence of fractures within the 3 groups is shown in this Kaplan-Meier plot. We observed a total of 501 fracture events. There were 158 events in sodium-glucose cotransporter–2 inhibitor (SGLT-2i) users (incidence ratio [IR], 4.69 fractures per 1000 person-years) compared with 195 in dipeptidyl peptidase 4 inhibitor (DPP-4i) users (IR, 5.26 fractures per 1000 person-years) and 148 in glucagon-like peptide 1 receptor agonist (GLP-1RA) users (IR, 4.71 fractures per 1000 person-years). SGLT-2i use was not with associated fracture compared with DPP-4i (hazard ratio, 0.90; 95% CI, 0.73-1.11) or GLP-1RA use (hazard ratio, 1.00; 95% CI, 0.80-1.25).

Across unmatched groups, DPP-4i users had the highest IR of fractures (7.55 fractures per 1000 person-years), followed by GLP-1RA users (IR, 4.76 fractures per 1000 person-years), and SGLT-2i users (IR, 4.36 fractures per 1000 person-years), resulting in a decreased risk of fracture associated with the use of SGLT-2i compared with DPP-4i (HR, 0.59; 95% CI, 0.51-0.68) and a similar risk compared with GLP-1RA (HR, 0.92; 95% CI, 0.76-1.12) (eTable 5 in the Supplement). After matching, we observed a total of 501 fracture events. There were 158 events in SGLT-2i users (IR, 4.69 fractures per 1000 person-years) compared with 195 events in DPP-4i users (IR, 5.26 fractures per 1000 person-years) and 148 in GLP-1RA users (IR, 4.71 fractures per 1000 person-years). There was no difference in the risk of fracture in SGLT-2i users compared with DPP-4i users (HR, 0.90; 95% CI, 0.73-1.11) or GLP-1RA users (HR, 1.00; 95% CI, 0.80-1.25) (Table 2). The cumulative incidence of fractures within the 3 groups is shown in a Kaplan-Meier plot (Figure 2).

**Table 2.** Number of Events, IRs, and HRs for Outcomes in 3-Way Propensity Score–Matched Groups

Outcome	Events, No. (IR, fractures/1000 PY)		SGLT-2i vs DPP-4i, HR (95% CI)	GLP-1RA, events, No. (IR, fractures/1000 PY) (n = 45 889)	SGLT-2i vs GLP-1RA, HR (95% CI)
	SGLT-2i (exposure) (n = 45 889)	DPP-4i (referent) (n = 45 889)			
Primary outcome, fracture	158 (4.69)	195 (5.26)	0.90 (0.73-1.11)	148 (4.71)	1.00 (0.80-1.25)
Secondary outcomes					
Falls	1666 (50.83)	2212 (61.95)	0.82 (0.77-0.87)	1617 (52.79)	0.96 (0.90-1.03)
Hypoglycemia	529 (15.78)	768 (20.90)	0.75 (0.67-0.84)	557 (17.82)	0.90 (0.79-1.01)
Syncope	372 (11.09)	424 (11.50)	0.95 (0.83-1.09)	394 (12.62)	0.89 (0.78-1.03)
Control outcomes					
Diabetic ketoacidosis	96 (2.85)	80 (2.15)	1.29 (0.96-1.74)	58 (1.84)	1.58 (1.14-2.18)
Heart failure hospitalization	280 (8.32)	723 (19.65)	0.42 (0.37-0.48)	379 (12.09)	0.69 (0.59-0.80)

Abbreviations: DPP-4i, dipeptidyl peptidase inhibitors; GLP-1RA, glucagon-like peptide-1 receptor agonists; HR, hazard ratio; IR, incidence rate; PY, person-years; SGLT-2i, sodium-glucose cotransporter–2 inhibitors.

### Sensitivity and Subgroup Analyses

**Figure 3.**
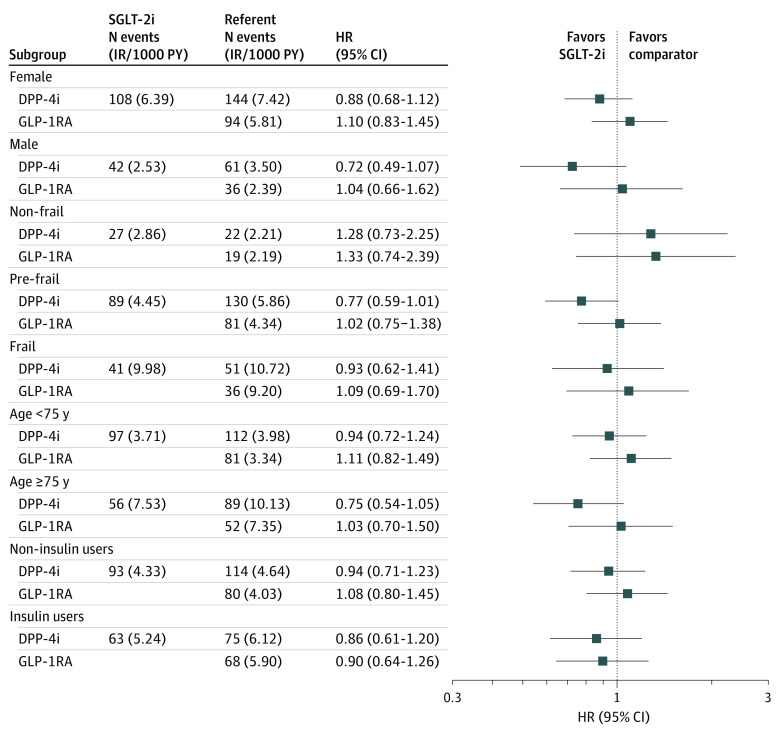
Forest Plot of Subgroup Analyses for Matched Fracture Outcome We tested for the presence of effect modification in 3 relevant subgroups: (1) nonfrail, prefrail, and frail patients; (2) patients aged less than 75 years vs 75 years and older, and (3) baseline insulin users vs nonusers. The incidence rate (IR) of fracture increased with frailty, older age, and insulin use; there was no evidence of effect modification on the fracture outcome based on frailty status, age less than 75 years vs 75 years and older, or insulin use. DPP-4i indicates dipeptidyl peptidase 4 inhibitor; GLP-1RA, glucagon-like peptide 1 receptor agonist; HR, hazard ratio; SGLT-2i, sodium-glucose cotransporter–2 inhibitor; PY, person-years.

When we adjusted for death as a competing risk for fracture using the Fine and Gray method,^38,39^ the matched results were unchanged (SGLT-2i vs DPP-4i, HR, 0.90 [95% CI, 0.73-1.11]; SGLT-2i vs GLP-1RA, HR, 1.00 [95% CI, 0.78-1.25]). Changing the grace period, carrying the index exposure forward, and limiting the analysis to canagliflozin users only produced consistent results (eTable 6 in the Supplement). The rate of fracture increased with female sex, frailty, older age, and insulin use; there was no evidence of effect modification on the fracture outcome based on sex, frailty status, age less than 75 vs 75 years or older, or insulin use (Figure 3).

### Secondary Outcomes

The risk of falls and hypoglycemia were lower in SGLT-2i users compared with matched DPP-4i users (HR, 0.82 [95% CI, 0.77-0.87] vs 0.75 [95% CI, 0.67-0.84]); there was no difference in syncope. There were no differences in falls, hypoglycemia, or syncope for SGLT-2i users compared with GLP-1RA users (Table 2).

### Validation of Findings Against Control Outcomes

Consistent with previous knowledge on the safety of SGLT-2i,^24,25^ we observed a higher risk of diabetic ketoacidosis associated with initiating SGLT-2i compared with initiating DPP-4i (HR, 1.29; 95% CI, 0.96-1.74) or GLP-1RA (HR, 1.58; 95% CI, 1.14-2.18). Similarly, we replicated the known association of SGLT-2i with a lower risk of heart failure hospitalization,^26,27,28^ compared with DPP-4i (HR, 0.42; 95% CI, 0.37-0.48) or GLP-1RA (HR, 0.69; 95% CI, 0.59-0.80).

## Discussion

In this nationwide cohort study using Medicare claims data, we found a similar risk of nontraumatic fracture in SGLT-2i users compared with matched DPP-4i users (HR, 0.90; 95% CI, 0.73-1.11) and GLP-1RA users (HR, 1.00; 95% CI, 0.80-1.25). Study findings were consistent across a range of predefined sensitivity and subgroup analyses.

Although SGLT-2i have shown prominent cardioprotective and nephroprotective effects compared with placebo,^2,3,4,6^ there was a concern that SGLT-2i may harm bone metabolism through modulating calcium and phosphate homeostasis,^16^ as well as the effects on weight loss.^40,41,42^ Conversely, it has been postulated that DPP-4i and GLP-1RA might have beneficial effects on bone health by promoting osteoblast differentiation and inhibiting osteoclast activity.^43,44,45^ Although the adverse effects of SGLT-2i on bone health are biologically plausible, clinical studies on fracture risk are inconsistent. In the CANVAS trial,^3^ the rate of all fractures was 26% higher with canagliflozin treatment than with placebo (HR 1.26; 95% CI, 1.04-1.52). Most fractures were low-trauma fractures and were balanced between the upper and lower limbs,^3,46^ and there was a higher fracture rate in female compared with male participants,^46^ which is consistent with our results. As a result of the interim results of CANVAS, the US Food and Drug Administration issued the warning for canagliflozin related to the increased risk of bone fractures in 2015.^7^ However, there was no definitive explanation for the increased fracture risk in CANVAS.^47^ This increased fracture risk was not observed in the following Canagliflozin and Renal Outcomes in Type 2 Diabetes and Nephropathy trial and other large RCTs,^2,4,5,6,17^ nor was an association observed in subsequent meta-analyses.^48,49^ A disproportionality and Bayesian analysis of Food and Drug Administration safety reporting data from 2004 to 2019 also showed no difference in fracture event reports for patients taking SGLT-2i vs SGLT-2i plus other diabetes agents.^50^ Thus, cohort studies to assess the effect of SGLT-2i on fracture in routine practice are warranted.

Previous studies showed that use of SGLT-2i or canagliflozin was not associated with an increased risk of fracture compared with DPP-4i (HR, 1.11; 95% CI, 0.96-1.28)^51^ or GLP-1RA (HR, 0.98 [95% CI, 0.75-1.26]^22^ and 1.11 [95% CI, 0.93-1.33]^52^) in relatively young populations (mean ages, 55-61 years). We used Medicare claims data, which collect health care information on the vast majority of legal US residents aged 65 years and older, to provide real-world evidence on the association of SGLT-2i fractures among older adults: our study yielded results consistent with these previous findings.^53^

We used 1:1:1 matching to identify older patients who were likely to receive either an SGLT-2i, DPP-4i, or GLP-1RA as add-on therapy for T2D based on individual covariates. Previous studies included predominantly younger patients and did not account for frailty in the analyses. We found no difference in fracture in those aged 65 to 74 years vs those aged 75 years and older. Using a validated frailty index,^30,31,32,33^ we found no fracture association in new users of SGLT-2i with or without markers of frailty. We also conducted prespecified secondary analyses to elucidate whether the use of SGLT-2i was associated with factors potentially related to fractures (ie, falls, hypoglycemia, or syncope). There were no differences in falls, hypoglycemia, or syncope for SGLT-2i users compared with GLP-1RA users. SGLT-2i were associated with a decreased risk of falls and hypoglycemia compared with matched DPP-4i, but there was no difference in syncope. Before matching, DPP-4i initiators were older and frailer compared with SGLT-2i initiators; thus, even though we adjusted for many measured factors including age and frailty status, residual confounding due to unmeasured factors, such as mild cognitive impairment is possible. Further studies may also clarify whether the initiation of SGLT-2i may be followed by adjustment in concomitant medications (eg, deprescribing diuretics or insulin),^54^ which might be associated with a reduced risk of falls and hypoglycemia events.

### Limitations

Our study has several limitations. First, considering the nature of observational studies, residual confounding by unmeasured factors cannot be ruled out. For instance, our Medicare data set had no information on relevant clinical variables including duration of diabetes, hemoglobin A_1c_ values, vitamin D and parathyroid hormone levels, and body mass index. This limited our ability to adjust for diabetes severity, glycemic control, and bone health. However, propensity score methods like ours may balance unmeasured characteristics, including diabetes duration and body mass index.^55^ In addition, our study was able to replicate the known associations of SGLT-2i with an increased risk of diabetic ketoacidosis and with a reduction in the risk of hospitalization for heart failure, providing further reassurance with respect to the validity of our findings.^25,27,28,56^ We were not able to evaluate the long-term effects of SGLT-2i on bone health given the short duration of follow-up (<1 year for the primary analysis). However, in the CANVAS trial, fracture events occurred as early as 12 weeks after treatment.^41^ We also excluded patients with previous fractures, which may limit the generalizability of our findings to older adults with previous fractures. Furthermore, our propensity score match may have ultimately excluded patients who were at the highest risk for fracture, as the analysis retained patients initiating DPP-4i or GLP1-RA who were more similar to the patients initiating SGLT-2i (ie, those who were younger, with fewer comorbid conditions, and less likely to be frail). Thus, our findings may not be generalizable to individuals at the highest risk for fracture, which is an area for future study.

## Conclusions

In this nationwide propensity score–matched Medicare cohort of older adults with T2D, the use of SGLT-2i was not associated with an increased risk of nontraumatic fractures compared with DPP-4i or GLP-1RA. Results were consistent across categories of sex, frailty, age, and insulin use. Our results add to the evidence base evaluating the safety profile of SGLT-2i in older adults outside of RCTs and further characterize the risk-benefit balance of SGLT-2i in clinical practice.

## Notes

Supplement. **eTable 1.** Definition of Inclusion and Exclusion Criteria **eTable 2. **Definition of Outcomes **eTable 3.** Full Baseline Characteristics in the SGLT-2i, DPP-4i, and GLP-1RA Groups Before and After Propensity Score Matching **eTable 4.** Censoring Reasons and Duration of Follow-up for Outcomes in Matched Groups **eTable 5.** Number of Events, Incidence Rates, and Hazard Ratios for Outcomes in Unmatched Groups **eTable 6.** Number of Events, Incidence Rates, and Hazard Ratios for Sensitivity Analyses in 3-Way PS-Matched Groups
