# Supplementary material for: Association of Sodium-Glucose Cotransporter–2 Inhibitors With Fracture Risk in Older Adults With Type 2 Diabetes
Source: JAMA Netw Open. 2021 Oct 27;4(10):e2130762. doi: 10.1001/jamanetworkopen.2021.30762 (PMC8552056; doi:10.1001/jamanetworkopen.2021.30762)
Supplement: Supplement. — eTable 1. Definition of Inclusion and Exclusion Criteria eTable 2. Definition of Outcomes eTable 3. Full Baseline Characteristics in the SGLT-2i, DPP-4i, and GLP-1RA Groups Before and After Propensity Score Matching eTable 4. Censoring Reasons and Duration of Follow-up for Outcomes in Matched Groups eTable 5. Number of Events, Incidence Rates, and Hazard Ratios for Outcomes in Unmatched Groups eTable 6. Number of Events, Incidence Rates, and Hazard Ratios for Sensitivity Analyses in 3-Way PS-Matched Groups [file jamanetwopen-e2130762-s001.pdf]

Supplemental Online Content

Zhuo M, Hawley CE, Paik JM, et al. Association of sodium-glucose cotransporter-2 inhibitors with fracture risk in older adults with type 2 diabetes. *JAMA Netw Open*. 2021;4(10):e2130762. doi:10.1001/jamanetworkopen.2021.30762

eTable 1. Definition of Inclusion and Exclusion Criteria
eTable 2. Definition of Outcomes
eTable 3. Full Baseline Characteristics in the SGLT-2i, DPP-4i, and GLP-1RA Groups Before and After Propensity Score Matching
eTable 4. Censoring Reasons and Duration of Follow-up for Fracture Outcome in Matched Groups
eTable 5. Number of Events, Incidence Rates, and Hazard Ratios for Outcomes in Unmatched Groups
eTable 6. Number of Events, Incidence Rates, and Hazard Ratios for Sensitivity Analyses in 3-Way PS-Matched Groups

This supplemental material has been provided by the authors to give readers additional information about their work.

© 2021 Zhuo M et al. *JAMA Network Open.*

eTable 1. Definition of Inclusion and Exclusion Criteria

Inclusion/exclusion criteria	Codes	Care setting	Position
Type 2 diabetes	ICD-9 DX: 250.*0, 250.*2<br>ICD-10 DX: E11.***	Inpatient, outpatient, carrier claims	Any
Type 1 diabetes	ICD-9 DX: 250.*1, 250.*3<br>ICD-10 DX: E10.***	Inpatient, outpatient, carrier claims	Any
Non-skin cancer	ICD-9 DX: 140.**-208.** (except 173.**)<br>ICD-10 DX: C00.**-C96.** (except C44.**), D03.**, D45	Inpatient, outpatient, carrier claims	Any
Human immunodeficiency virus	ICD-9 DX: 042, 079.53, V08<br>ICD-10 DX: B20, B97.35, Z21<br>Prescriptions: Abacavir, Amprenavir, Atazanavir, Darunavir, Delavirdine, Didanosine, Efavirenz, Emtricitabine, Enfuvirtide, Etravirine, Fosamprenavir, Indinavir, Lamivudine-Zidovudine, Maraviroc, Nelfinavir, Nevirapine, Raltegravir, Rilpivirine, Ritonavir, Ritonavir-Lopinavir, Saquinavir, Stavudine, Tipranavir, Zalcitabine, Zidovudine		
End stage kidney disease	Occurrence of two of the following ESRD/Dialysis codes at least 30 days apart -<br>ICD-9 DX: 585.5, 585.6, V56.8, V45.1, V56.0<br>ICD-10 DX: N18.5, N18.6, Z49.31, Z49.32, Z99.2<br>ICD-9 PX: 39.95, 54.98<br>ICD-10 PX: 3E1M39Z, 5A1D00Z, 5A1D60Z<br>CPT-4/HCPCS (Inpatient care setting only):<br>50360,50365, 50380, 90920, 90921, 90924, 90925, 90935,90937, 90940, 90945, 90947, 90957, 90958, 90959,<br>90960, 90961, 90962, 90965, 90966, 90969, 90970, 90989, 90993, 90999, 99512, 99559, G0257, G0314, G0315, G0316, G0317, G0318, G0319, G0322, G0323,<br>G0326, G0327, S9335, S9339	Inpatient, outpatient, carrier claims	Any

© 2021 Zhuo M et al. *JAMA Network Open.*

eTable 2. Definition of Outcomes

Outcome	Codes		Care setting	Position
Fracture	Occurrence of a fracture of the Humerus, Wrist, Hip, or Pelvis without the occurrence of Injury by external cause		-	-
	Humerus	Occurrence of one diagnosis and one procedure code within 30 days –		
		Diagnosis Codes:<br>ICD-9 DX: 812.*, 733.11<br>ICD-10 DX: M80.02*A, M80.82*A, M84.42*A, S42.2**A, S42.2**B, S42.3**A, S42.3**B, S42.4**A, S42.4**B, S42.9*XA, S42.9*XB, S49.***A	Inpatient, outpatient, carrier claims	Any
		Procedure Codes:<br>ICD-9 PX: 79.01, 78.52, 79.31, 79.61, 79.11, 79.21<br>ICD-10 PX: 0PHC04Z, 0PHC06Z, 0PHC34Z, 0PHC36Z, 0PHC44Z, 0PHC46Z, 0PHD04Z, 0PHD06Z, 0PHD34Z, 0PHD36Z, 0PHD44Z, 0PHD46Z, 0PHF04Z, 0PHF06Z, 0PHF34Z, 0PHF36Z, 0PHF44Z, 0PHF46Z, 0PHG04Z, 0PHG06Z, 0PHG34Z, 0PHG36Z, 0PHG44Z, 0PHG46Z<br>0PSC*, 0PSD*, 0PSF*, 0PSG*<br>HCPCS: 24586, 24587, 23600, 23615, 23620, 23625, 23630, 23665, 23680, 24500, 24505, 24515, 24516, 24530, 24535, 24538, 24560, 24575, 23605, 23670, 24540, 24545, 24565	Inpatient, outpatient	Any
	Wrist	Occurrence of one diagnosis and one procedure code within 30 days –		
		Diagnosis Codes:<br>ICD-9 DX: 733.12, 813.*<br>ICD-10 DX: M80.03*A, M80.83*A, M84.43*A, M84.63*A, S52.*A, S52.*B, S52.*C, S59.*A (except S59.8**A, S59.9**A)	Inpatient, outpatient, carrier claims	Any
		Procedure Codes:<br>ICD-9 PX: 79.22, 79.62, 78.53, 79.02, 79.12, 79.32<br>ICD-10 PX: 0PHH04Z, 0PHH06Z, 0PHH34Z, 0PHH36Z, 0PHH44Z, 0PHH46Z, 0PHJ04Z, 0PHJ06Z, 0PHH34Z, 0PHJ36Z, 0PHJ44Z, 0PHJ46Z, 0PHK04Z, 0PHK06Z, 0PHK34Z, 0PHK36Z, 0PHK44Z, 0PHK46Z, 0PHL04Z, 0PHL06Z, 0PHL34Z, 0PHL36Z, 0PHL44Z, 0PHL46Z, 0PSH*, 0PSJ*, 0PSK*, 0PSL*<br>HCPCS: 24665, 24666, 25611, 24620, 24635, 24650, 24655, 25500, 25530, 25545, 25565,	Inpatient, outpatient	Any

© 2021 Zhuo M et al. *JAMA Network Open.*

		25620, 25650, 24670, 24685, 25505, 25515, 25535, 25560, 25575, 25600, 25605		
	Hip	Occurrence of one diagnosis and one procedure code within 30 days –		
		Diagnosis Codes:<br>ICD-9 DX: 733.14, 733.96, 733.97, 820.*<br>ICD-10 DX: M80.05*A, M80.85*A, M84.35*A (except M84.350A, M84.354A), M84.45*A (except M84.450A, M84.454A), M84.65*A (except M84.650A, M84.654A), M84.45*A, S72.*A, S72.*B, S72.*C, S79.*A (except S79.8*A, S79.9*A)	Inpatient	Any
		Procedure Codes:<br>ICD-9 PX: 79.15, 79.05, 79.35, 78.55, 79.25, 79.65<br>ICD-10 PX: 0QH604Z, 0QH606Z, 0QH634Z, 0QH636Z, 0QH644Z, 0QH646Z, 0QH704Z, 0QH706Z, 0QH734Z, 0QH736Z, 0QH744Z, 0QH746Z, 0QH804Z, 0QH806Z, 0QH834Z, 0QH836Z, 0QH844Z, 0QH846Z, 0QH904Z, 0QH906Z, 0QH934Z, 0QH936Z, 0QH944Z, 0QH946Z, 0QHB04Z, 0QHB06Z, 0QHB34Z, 0QHB36Z, 0QHB44Z, 0QHB46Z, 0QHC04Z, 0QHC06Z, 0QHC34Z, 0QHC36Z, 0QHC44Z, 0QHC46Z, 0QS6*, 0QS7*, 0QS8*, 0QS9*, 0QSB*, 0QSC*<br>HCPCS: 27238, 27240, 27267, 27232, 27235, 27125, 27236, 27268, 27130, 27230, 27244, 27245, 27246, 27248, 27269	Inpatient	Any
	Pelvis	Occurrence of one diagnosis or one procedure code		
		Diagnosis Codes:<br>ICD-9 DX: 733.98, 808.*<br>ICD-10 DX: M84.350A, S32.3*A, S32.3*B, S32.4*A, S32.4*B, S32.5*A, S32.5*B, S32.6*A, S32.6*B, S32.8*A, S32.8*B, S32.9*A, S32.9*B	Inpatient, outpatient, carrier claims	Any
		Procedure Codes:<br>HCPCS: 27193, 27215, 27216, 27217, 27220, 27226, 27227, 27194, 27200, 27202, 27218, 27222, 27228	Inpatient, outpatient	Any
	Injury by External Cause	ICD-9 DX: E002.*, E004.*, E016.*, E017.*, E800.*-E807.*, E808.9, E810.*-E838.* (except E839.*), E881.*-E886.* (except E885.*), E916, E917.*, E919.*, E957.*, E987.*<br>ICD-10 DX: V00-V99	Inpatient, Outpatient, carrier claims	Any
Falls	ICD-9 DX: E880.* E881.* E882 E883.* E884.* E885.* E886.* E887 E888.*		Inpatient, outpatient,	Any

© 2021 Zhuo M et al. *JAMA Network Open.*

	ICD-10 DX: V00.* (except V00.0*, V00.84*, V00.*S), W00.*-W19.* (except W00.*S-W19.*S)	carrier claims	
Hypoglycemia	ICD-9 DX: 962.3, 251.0, 251.1, 251.2<br>ICD-10 DX: E16.0, E16.1, E16.2, E08.649, E10.649, E11.649, E13.649, E*.641<br><br>Emergency Room care setting was defined as the following:<br>Carrier Claims Line Place of Service Code: 23<br>Outpatient Revenue Center Code: 0451, 0452, 0459, 0450, 0456	Inpatient, Emergency Room	Inpatient Primary, Any Emergency Room
Syncope	ICD-9 DX: 780.2<br>ICD-10 DX: R55	Inpatient, outpatient, carrier claims	Any

© 2021 Zhuo M et al. *JAMA Network Open.*

eTable 3. Full Baseline Characteristics in the SGLT-2i, DPP-4i, and GLP-1RA Groups Before and After Propensity Score Matching

	Unmatched			3-Way PS-Matched		
**Baseline characteristics, n (%)**	**SGLT-2i (N = 62,454)**	**DPP-4i (N = 338,463)**	**GLP-1RA (N = 66,016)**	**SGLT-2i* (N = 45,889)**	**DPP-4i (N = 45,889)**	**GLP-1RA (N = 45,889)**
**Demographics**						
Age; mean (sd)	71.94(5.17)	74.69(6.71)	71.46(4.84)	71.60(4.96)	71.64(5.13)	71.67(4.97)
Male	31796(50.91)	147777(43.66)	29064(44.03)	21548(46.96)	21053(45.88)	21525(46.9)
Race/ethnicity						
White	51420(82.33)	254238(75.12)	55630(84.27)	38626(84.17)	38704(84.34)	38455(83.80)
Black	4577(7.33)	35969(10.63)	5515(8.35)	3534(7.70)	3484(7.59)	3586(7.81)
Other	6457(10.34)	48256(14.26)	4871(7.38)	3729(8.13)	3701(8.07)	3848(8.39)
Region						
Midwest	12732(20.39)	70357(20.79)	15625(23.67)	10197(22.22)	10215(22.26)	10109(22.0)
Northeast	10715(17.16)	63919(18.89)	10113(15.32)	7330(15.97)	7158(15.60)	7349(16.01)
South	28117(45.02)	143629(42.44)	28648(43.40)	20391(44.44)	20541(44.76)	20416(44.4)
West	10890(17.44)	60558(17.89)	11630(17.62)	7971(17.37)	7975(17.38)	8015(17.47)
**Diabetes-related conditions**						
Diabetic nephropathy	6488(10.39)	48370(14.29)	12119(18.36)	5944(12.95)	6038(13.16)	6105(13.30)
Diabetic neuropathy	15808(25.31)	79491(23.49)	21058(31.90)	12859(28.02)	13111(28.57)	12966(28.2)
Diabetic retinopathy	6513(10.43)	32838(9.70)	8972(13.59)	5294(11.54)	5306(11.56)	5323(11.60)
Endocrinologist visit during prior 365 days	10894(17.44)	44589(13.17)	18034(27.32)	9732(21.21)	9541(20.79)	9960(21.70)
HbA1c tests ordered during prior 365 days, count; mean (sd)	2.75(1.33)	2.60(1.40)	2.84(1.41)	2.79(1.35)	2.78(1.39)	2.77(1.36)
Hypoglycemia	4871(7.80)	26451(7.82)	6342(9.61)	3845(8.38)	3921(8.54)	3974(8.66)
**Comorbid conditions**						
Heart failure	7037(11.27)	55737(16.47)	10235(15.50)	5825(12.69)	5965(13.00)	5979(13.03)
Hypertension	57598(92.22)	315528(93.22)	61841(93.68)	42626(92.89)	42657(92.96)	42650(92.9)
Ischemic heart disease	21178(33.91)	120679(35.66)	23866(36.15)	15834(34.51)	15788(34.40)	15914(34.6)
Ischemic or hemorrhagic stroke	7231(11.58)	47627(14.07)	8116(12.29)	5418(11.81)	5504(11.99)	5429(11.83)
Renal disease (non-diabetic)	11987(19.19)	107932(31.89)	20177(30.56)	10848(23.64)	10955(23.87)	10920(23.80)
**Falls or Fracture-related conditions**						
Bone mineral density screening	4864(7.79)	27645(8.17)	5941(9.00)	3916(8.53)	3939(8.58)	3911(8.52)
Dementia	3573(5.72)	35947(10.62)	4023(6.09)	2664(5.81)	2856(6.22)	2735(5.96)
Falls or syncope	3373(5.40)	26349(7.78)	4489(6.80)	2777(6.05)	2837(6.18)	2777(6.05)
Frailty category[1]						
Non-frail: <0.15	19689(31.53)	82847(24.48)	14905(22.58)	12319(26.85)	11976(26.10)	12183(26.5)
Pre-frail: 0.15-0.24	36114(57.82)	195611(57.79)	40343(61.11)	27818(60.62)	27940(60.89)	27779(60.5)
Frail: ≥0.25	6651(10.65)	60005(17.73)	10768(16.31)	5752(12.53)	5973(13.02)	5927(12.92)

© 2021 Zhuo M et al. *JAMA Network Open.*

Glaucoma or cataracts	27040(43.30)	140445(41.49)	28931(43.82)	19989(43.56)	19997(43.58)	19965(43.51)
Mobility limitations	1726(2.76)	15564(4.60)	2461(3.73)	1379(3.01)	1433(3.12)	1433(3.12)
Osteoporosis	4458(7.14)	33373(9.86)	4932(7.47)	3305(7.20)	3375(7.35)	3402(7.41)
**Other comorbid conditions**						
Delirium/Psychosis	2468(3.95)	22128(6.54)	3210(4.86)	1989(4.33)	2067(4.50)	2027(4.42)
Peripheral arterial disease or surgery	7622(12.20)	52493(15.51)	9256(14.02)	5854(12.76)	5840(12.73)	5936(12.94)
**Falls or Fracture-related medications**						
ACE inhibitors or ARBs	48725(78.02)	261515(77.27)	52582(79.65)	36259(79.01)	36190(78.86)	36234(78.96)
Anticholinergics	12271(19.65)	73955(21.85)	15032(22.77)	9687(21.11)	9901(21.58)	9679(21.09)
Anticonvulsants	12586(20.15)	67714(20.01)	16649(25.22)	10331(22.51)	10646(23.20)	10362(22.5)
Antidepressants	17786(28.48)	93185(27.53)	23568(35.70)	14854(32.37)	15178(33.08)	15055(32.8)
Benzodiazepines	5205(8.33)	30516(9.02)	6051(9.17)	4087(8.91)	4060(8.85)	22863(49.8)
Beta blockers	30249(48.43)	175935(51.98)	34252(51.88)	22732(49.54)	22791(49.67)	15589(33.9)
Calcium channel blockers	20700(33.14)	128587(37.99)	23149(35.07)	15595(33.98)	15450(33.67)	10029(21.85)
Diuretics, loop	11326(18.13)	77999(23.05)	17758(26.90)	9789(21.33)	10081(21.97)	9997 (21.77)
Diuretics, thiazide	9860(15.79)	56419(16.67)	12036(18.23)	7751(16.89)	7920(17.26)	7833(17.07)
Diuretics, other	2614(4.19)	16240(4.80)	3997(6.05)	2262(4.93)	2284(4.98)	2297(5.01)
Nitrates	5859(9.38)	36423(10.76)	7089(10.74)	4470(9.74)	4425(9.64)	4500(9.81)
Opioids	9217(14.76)	55285(16.33)	12474(18.90)	7608(16.58)	7830(17.06)	7730(16.84)
Osteoporosis medications[2]	2445(3.91)	20643(6.10)	2483(3.76)	1718(3.74)	1761(3.84)	1735(3.78)
Sedative hypnotics[3]	2452(3.93)	14485(4.28)	3100(4.70)	1970(4.29)	1909(4.16)	1965(4.28)
Oral steroids	11763(18.83)	65136(19.24)	13447(20.37)	8966(19.54)	8943(19.49)	9081(19.79)
Total number of medications; mean (sd)	12.92(5.82)	13.13(6.10)	14.54(6.19)	13.60(5.99)	13.71(6.14)	13.72(5.82)
**Diabetes medications**						
Number of diabetes drugs; mean (sd)	2.34(0.80)	2.18(0.77)	2.32(0.82)	2.32(0.81)	2.33(0.81)	2.33(0.82)
Insulin	17492(28.01)	52226(15.43)	29693(44.98)	16312(35.55)	16234(35.38)	16403(35.74)
Metformin	48832(78.19)	247812(73.22)	44883(67.99)	33971(74.03)	34081(74.27)	33932(73.94)
Sulfonylureas	29342(46.98)	164362(48.56)	27694(41.95)	20083(43.76)	20746(45.21)	20614(44.92)
Thiazolidinediones	6766(10.83)	29792(8.80)	6547(9.92)	4682(10.20)	4755(10.36)	4868(10.61)
**Other medications**						
Anti-arrhythmics	1320(2.11)	9977(2.95)	1703(2.58)	1042(2.27)	1081(2.36)	1059(2.31)
Anticoagulants	6015(9.63)	38537(11.39)	7265(11.00)	4637(10.10)	4638(10.11)	4729(10.31)
Antiparkinsonian medications	2213(3.54)	12616(3.73)	3143(4.76)	1881(4.10)	1932(4.21)	1916(4.18)
Antiplatelets	10382(16.62)	60435(17.86)	11348(17.19)	7558(16.47)	7683(16.74)	7673(16.72)
Digoxin	1740(2.79)	12997(3.84)	1683(2.55)	1137(2.48)	1138(2.48)	1182(2.58)
Other hypertension medications	4964(7.95)	34998(10.34)	6597(9.99)	3971(8.65)	3989(8.69)	4073 (8.87)
**Healthcare utilization**						
Emergency department visits during prior 365 days	16498(26.42)	114540(33.84)	20435(30.95)	12996(28.32)	13263(28.90)	13152 (28.64)

© 2021 Zhuo M et al. *JAMA Network Open.*

Hospitalization during prior 365 days	7401(11.85)	62839(18.57)	9829(14.89)	5930(12.92)	6165(13.43)	6072 (13.22)
Office visits during prior 365 days; mean (sd)	10.88(7.55)	11.18(8.20)	12.51(8.56)	11.53(7.85)	11.58(8.27)	4028(8.78)
Pneumonia or influenza vaccine during prior 365 days; mean (sd)	1520 (2.43)	14952 (4.41)	2050 (3.10)	1160 (2.53)	1196 (2.60)	1237 (2.69)
**Lifestyle factors**						
Obesity	15762(25.24)	66432(19.63)	23242(35.21)	13666(29.78)	13781(30.03)	13822(30.12)
Smoking	11682(18.70)	63139(18.65)	13587(20.58)	9051(19.72)	9090(19.81)	9118(19.87)
**Year of Cohort Entry**						
2013	1097(1.76)	52910(15.63)	7043(10.67)	1097(2.39)	1123(2.45)	1191(2.60)
2014	8487(13.59)	77927(23.02)	10811(16.38)	7620(16.61)	7218(15.73)	7255(15.81)
2015	17114(27.40)	76575(22.62)	13069(19.80)	10953(23.87)	10744(23.41)	10996(23.96)
2016	16157(25.87)	68539(20.25)	15388(23.31)	11745(25.59)	12010(26.17)	11910(25.95)
2017	19599(31.38)	62512(18.47)	19705(29.85)	14474(31.54)	14794(32.24)	14537(31.68)

*64% patients in the matched SGLT-2i group were new canagliflozin users

"All standardized differences between the 3 drugs in each polypharmacy group were <0.10 indicating well-balanced groups after propensity score matching

PS: propensity-score; SGLT2i: sodium-glucose cotransporter-2 inhibitors; DPP-4: dipeptidyl peptidase inhibitors; GLP-1RA: glucagon-like peptide-1 receptor agonists; sd: standard deviation; HbA1c: hemoglobin A1c; ACE inhibitors or ARBs: angiotensin converting enzyme inhibitors or angiotensin-receptor blockers.

1 Kim DH, Schneeweiss S, Glynn RJ. Comparing Approaches to Measure Frailty in Medicare Data: Deficit-Accumulation Frailty Index Versus Phenotypic Frailty. J Gerontol A Biol Sci Med Sci. 2018;73(7):989-990

2 Osteoporosis medications included bisphosphonates, calcitonin, denosumab, raloxifene, romosozumab, tamoxifen, and teriparatide.

3 Sedative hypnotics included buspirone, chloral hydrate, diphenhydramine, doxylamine, eszopiclone, hydroxyzine, mepbromate, zaleplon, and zolpidem.

© 2021 Zhuo M et al. *JAMA Network Open.*

eTable 4. Censoring Reasons and Duration of Follow-up for Fracture Outcome in Matched Groups

	SGLT-2i	DPP-4	GLP-1 RA
	N events	N events	N events
	**Outcome: Fracture**		
Death	453	852	456
Disenrollment	2,018	2,373	1,866
Addition of Exposure	5,419	3,404	3,216
Termination of Exposure	24,526	23,301	26,730
End of Study Period	13,315	15,765	13,473
Outcome	158	195	148
Follow up, days, mean (median)	268 (262)	295 (278)	250 (249)
	**Outcome: Falls**		
Death	394	759	398
Disenrollment	1,963	2,287	1,824
Addition of Exposure	5,301	3,310	3,160
Termination of Exposure	23,739	22,420	25,953
End of Study Period	12,826	14,901	12,937
Outcome	1,666	2,212	1,617
Follow up, days, mean (median)	261(256)	284 (270)	244 (243)
	**Outcome: Hypoglycemia**		
Death	430	785	429
Disenrollment	2,001	2,350	1,856
Addition of Exposure	5,397	3,378	3,194
Termination of Exposure	24,266	23,016	26,465
End of Study Period	13,266	15,592	13,378
Outcome	529	768	557
Follow up, days, mean (median)	267 (262)	292 (277)	249 (248)
	**Outcome: Syncope**		
Death	439	842	440
Disenrollment	2,011	2,353	1,863
Addition of Exposure	5,396	3,385	3,207
Termination of Exposure	24,368	23,166	26,567
End of Study Period	13,303	15,719	13,418
Outcome	372	424	394
Follow up, days, mean (median)	267 (262)	293 (277)	249 (247)
	**Outcome: Diabetic Ketoacidosis**		
Death	441	855	455
Disenrollment	2,020	2,374	1,873
Addition of Exposure	5,429	3,406	3,218
Termination of Exposure	24,538	23,344	26,773
End of Study Period	13,365	15,830	13,512
Outcome	96	80	58
Follow up, days, mean (median)	268 (263)	296 (279)	251 (249)
	**Outcome: Heart Failure Hospitalization**		
Death	424	750	414
Disenrollment	2,018	2,358	1,864
Addition of Exposure	5,413	3,391	3,206
Termination of Exposure	24,433	23,049	26,592
End of Study Period	13,321	15,618	13,434
Outcome	280	723	379
Follow up, days, mean (median)	268 (262)	293 (277)	250 (248)

© 2021 Zhuo M et al. *JAMA Network Open.*

	Outcome: Death		
Disenrollment	2,023	2,376	1,874
Addition of Exposure	5,435	3,407	3,221
Termination of Exposure	24,596	23,398	26,811
End of Study Period	13,375	15,839	13,519
Outcome	460	869	464
Follow up, days, mean (median)	268 (263)	296 (280)	251 (249)

© 2021 Zhuo M et al. *JAMA Network Open.*

eTable 5. Number of Events, Incidence Rates, and Hazard Ratios for Outcomes in Unmatched Groups

	SGLT-2i N = 62,454 (exposure)	DPP-4i N = 338,463 (referent)	SGLT-2i vs DPP-4i	GLP-1 RA N = 66,016 (referent)	SGLT-2i vs GLP-1RA
**Primary outcome**	**N events**	**N events**	**HR**	**N events**	**HR**
	**(IR/1000 PY)**	**(IR/1000 PY)**	**(95% CI)**	**(IR/1000 PY)**	**(95% CI)**
Fracture	201 (4.36)	2,486 (7.55)	0.59 (0.51-0.68)	223 (4.76)	0.92 (0.76-1.12)
**Secondary outcomes**					
Falls	2,103 (46.82)	21,252 (67.70)	0.69 (0.66-0.72)	2,555 (56.16)	0.83 (0.79-0.88)
Hypoglycemia	632 (13.78)	7,325 (22.46)	0.60 (0.55-0.65)	995 (21.39)	0.65 (0.59-0.72)
Syncope	456 (9.94)	5,719 (17.56)	0.53 (0.48-0.59)	684 (14.72)	0.68 (0.60-0.76)
Death	623 (13.5)	12,059 (36.44)	0.38 (0.35-0.41)	747 (15.90)	0.85 (0.77-0.95)
**Control outcomes**					
Diabetic Ketoacidosis	123 (2.67)	634 (1.92)	1.34 (1.10-1.63)	100 (2.13)	1.26 (0.96-1.64)
Heart Failure Hospitalization	339 (7.36)	8,241 (25.29)	0.28 (0.25-0.31)	706 (15.13)	0.49 (0.43-0.55)

eTable 6. Number of Events, Incidence Rates, and Hazard Ratios for Sensitivity Analyses in 3-Way PS-Matched Groups

	SGLT-2i N = 45,889 (exposure)	DPP-4i N = 45,889 (referent)	SGLT-2i vs DPP-4i	GLP-1 RA N = 45,889 (referent)	SGLT-2i vs GLP-1RA
**Outcome**	**N events**	**N events**	**HR**	**N events**	**HR**
	**(IR/1000 PY)**	**(IR/1000 PY)**	**(95% CI)**	**(IR/1000 PY)**	**(95% CI)**
Grace period 30 days	119 (4.38)	140 (4.63)	0.95 (0.74-1.21)	110 (4.63)	0.95 (0.73-1.23)
Grace period 90 days	176 (4.69)	224 (5.45)	0.87 (0.71-1.06)	179 (4.93)	0.95 (0.77-1.17)
First exposure carried forward	163 (4.76)	189 (5.32)	0.90 (0.73-1.11)	181 (5.19)	0.92 (0.74-1.13)
Canagliflozin users only	120 (4.91)	195 (5.26)	0.93 (0.74-1.17)	148 (4.71)	1.04 (0.81-1.32)

© 2021 Zhuo M et al. *JAMA Network Open.*
